# Single molecule real time sequencing in ADTKD-*MUC1* allows complete assembly of the VNTR and exact positioning of causative mutations

**DOI:** 10.1038/s41598-018-22428-0

**Published:** 2018-03-08

**Authors:** Andrea Wenzel, Janine Altmueller, Arif B. Ekici, Bernt Popp, Kurt Stueber, Holger Thiele, Alois Pannes, Simon Staubach, Eduardo Salido, Peter Nuernberg, Richard Reinhardt, André Reis, Patrick Rump, Franz-Georg Hanisch, Matthias T. F. Wolf, Michael Wiesener, Bruno Huettel, Bodo B. Beck

**Affiliations:** 1 Institute of Human Genetics, University Hospital of Cologne, Cologne, Germany; 2 Cologne Center for Genomics (CCG) and Center for Molecular Medicine Cologne (CMMC), University of Cologne, Cologne, Germany; 3 Institute of Human Genetics, Friedrich-Alexander-Universität Erlangen-Nürnberg (FAU), Erlangen, Germany; 4 The Max Planck-Genome-Centre Cologne (MP-GC), Max Planck Institute for Plant Breeding Research, Carl-von-Linné-Weg 10, Cologne, Germany; 5 Pathology Department Universidad de La Laguna, Hospital Universitario de Canarias Ofra s/n, La Laguna, 38320 Tenerife, Spain; 6 Department of Genetics, Clinical Genetics Section University Medical Center Groningen, 9700 RB Groningen, The Netherlands; 7 Institute of Biochemistry II, Medical Faculty, University of Cologne, Cologne, Germany; 8 Pediatric Nephrology, University of Texas Southwestern Medical Center, Dallas, TX USA; 9 Department of Nephrology and Hypertension, Friedrich-Alexander-Universität Erlangen-Nürnberg, Erlangen, Germany; 10 Unaffiliated, Huerth, Germany

## Abstract

Recently, the *Mucin-1* (*MUC1*) gene has been identified as a causal gene of autosomal dominant tubulointerstitial kidney disease (ADTKD). Most causative mutations are buried within a GC-rich 60 basepair variable number of tandem repeat (VNTR), which escapes identification by massive parallel sequencing methods due to the complexity of the VNTR. We established long read single molecule real time sequencing (SMRT) targeted to the *MUC1*-VNTR as an alternative strategy to the snapshot assay. Our approach allows complete VNTR assembly, thereby enabling the detection of all variants residing within the VNTR and simultaneous determination of VNTR length. We present high resolution data on the VNTR architecture for a cohort of snapshot positive (n = 9) and negative (n = 7) ADTKD families. By SMRT sequencing we could confirm the diagnosis in all previously tested cases, reconstruct both VNTR alleles and determine the exact position of the causative variant in eight of nine families. This study demonstrates that precise positioning of the causative mutation(s) and identification of other coding and noncoding sequence variants in ADTKD-*MUC1* is feasible. SMRT sequencing could provide a powerful tool to uncover potential factors encoded within the VNTR that associate with intra- and interfamilial phenotype variability of *MUC1* related kidney disease.

## Introduction

Autosomal dominant tubulointerstitial kidney disease (ADTKD; formerly known as medullary cystic kidney disease (MCKD)) constitutes a prototypic group of usually slower progressive nephropathies. ADTKD can manifest in childhood, but typically comes to medical attention with chronic kidney disease from early adulthood onwards^1–4^.

With the recently identified ADTKD-*MUC1* subtype (OMIM #174000; originally referred to as MCKD type 1), four genes (*UMOD* (OMIM #603860, #162000 and #609886; ADTKD-UMOD; MCKD type 2), *REN* (OMIM #613092; ADTKD-*REN*), and *HNF1B* (OMIM #137920; ADTKD-*HNF1B*)) have so far been repeatedly assigned to the ADTKD-spectrum^1,5–8^. Shared clinical features in ADTKD and phenotype overlap with other renal kidney diseases (RKDs) frequently make a definite clinical or histopathological diagnosis difficult^4,8,9^. Sanger sequencing or NGS based analyses of the first three identified genes is a widely available standard, while analysis of the *MUC1* gene still poses a substantial diagnostic problem. ADTKD-*MUC1* constitutes the first kidney disorder where mutations reside within a coding variable number of tandem repeat (VNTR contained in exon 2). The inaccessibility of many genomic repeats to direct short-read based sequencing approaches explains why the association of the *MUC1* gene to ADTKD has been obscured for long^5^.

**Figure 1:**
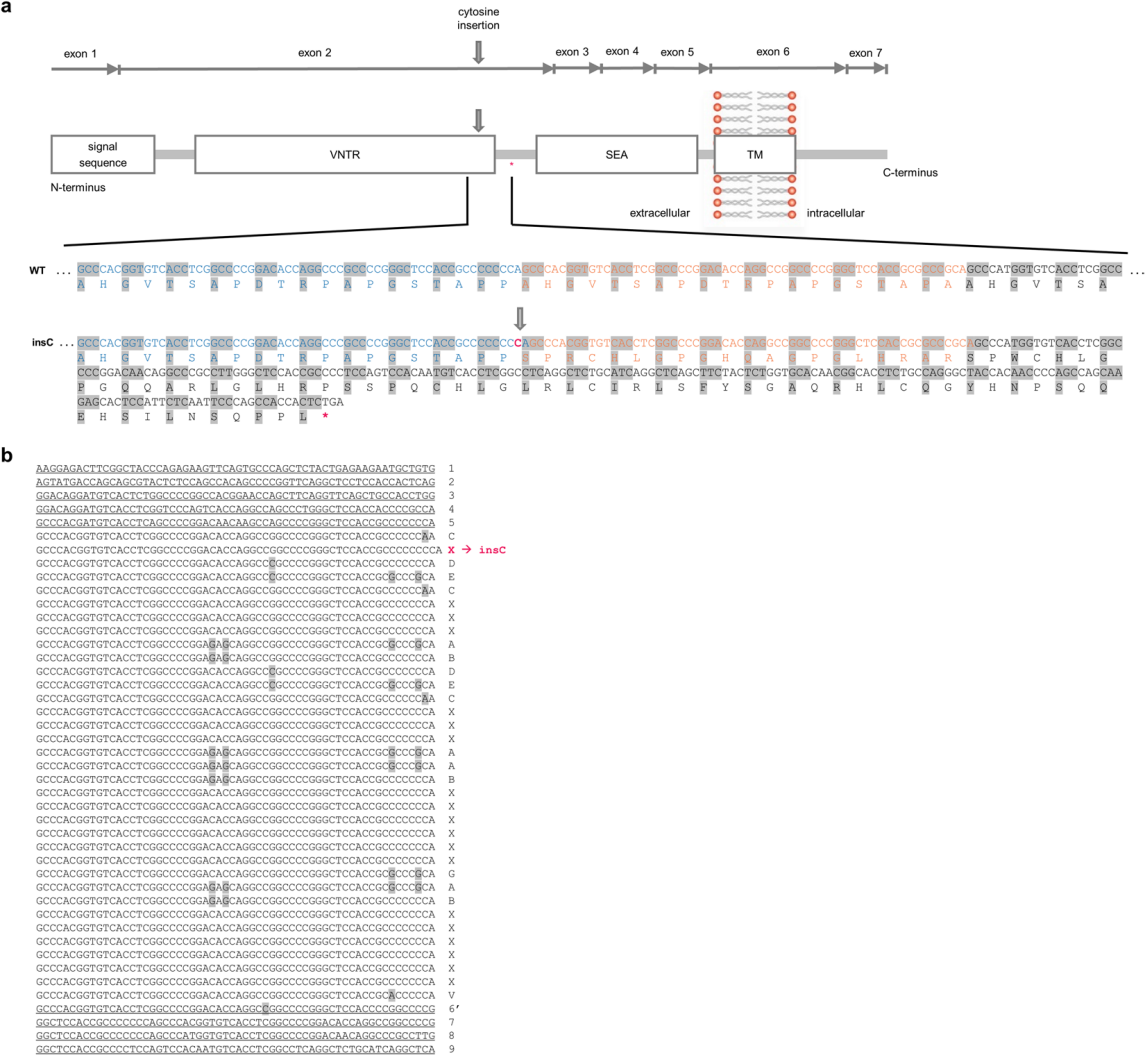
(**a**) Localization and consequences of the cytosine insertion in the VNTR domain of the *MUC1* gene. Exons 1–7 of the *MUC1* gene are shown on cDNA level (UniProt ID P15941). Domains of the corresponding full-length protein are displayed (TM, transmembrane domain; SEA, sea-urchin sperm protein, enterokinase and agrin-domain). The cytosine insertion into the 7C-homopolymer in a single repeat unit of the variable number of tandem repeats (VNTR) domain is exemplarily introduced into the next-to-last repeat unit (indicated by arrows). In the lower part, coding genomic VNTR sequence and the corresponding amino acid sequence are shown for WT and the insC mutant. The insC causes a frameshift introducing a premature stop codon shortly beyond the VNTR domain and thereby, creating a ‘neoprotein‘ lacking the SEA, TM and the cytoplasmic domains (**b**) *MUC1*-VNTR assembly of the risk allele in family F1. The complete VNTR sequence is shown for affected individuals III-10, IV-16, IV-27, and IV-28. This allele was identified in all affected individuals but it was not identified in unaffected individuals. The insC was detected in the second consensus repeat (highlighted in red). Capital letter code of the 60 mer VNTR-units according to Kirby *et al*. are displayed next to each repeat sequence. Nucleotide sequence differences within the repeat units are highlighted in dark grey. Uniform pseudo-repeats (nonvariable) units at beginning and at the end of the VNTR are underlined.

In contrast to simple tandem repeats commonly seen in (coding and noncoding) repeat expansion diseases (e.g. Huntington Disease or Fragile X Syndrome etc.) the actual size of the coding 60 basepair VNTR in ADTKD-*MUC1* seems irrelevant to the pathomechanism of ADTKD (Fig. 1)^5,10,11^. As the VNTR size of each parental *MUC1* allele is highly polymorphic (usually between 20 to 125 units per allele) with no expansion or retraction of units, commonly used methods to detect repeat size alterations are per se useless here^12^.

The prototypic causative variant in *MUC1* associated ADTKD, the insertion of a eighth cytosine base (insC) in a seven cytosine stretch within one unit of the VNTR composed of almost identical 60-mer units (according to HGVS nomenclature recommendations a duplication, but for the sake of clarity we stay with term insertion used in most publications), has been repeatedly found worldwide (also arbitrarily referred to as c.428dupC)^4,5^. Based on the VNTR assembly from three families, the exact location of the insC with regard to the unit number seemed to be variable, although always occurring within the 7C-homopolymer at relative base positions 53–59 of the 60-mer unit (Fig. 1)^5^. Due to the nature of the VNTR, insC within different located 60-mer unit numbers as well as other small insertions or deletions located at different positions, would result in a frameshift leading to a similar, presumably toxic MUC1-neoprotein that is retained within the cell (Fig. 1)^5^.

The signal transducing transmembrane MUC1 protein is abundantly expressed in most epithelia and over-expressed in epitheloid malignancies. MUC1 is also expressed in renal development and later found at the apical surfaces of normal kidney epithelia that form luminal surfaces^13–16^. The role of MUC1 in the kidney is not very clear apart from the N terminal ectodomain of the glycoprotein being released into the urine. At urothelial surfaces MUC1 and uromodulin form protective polymer layers. Recently it was shown that MUC1 regulates the renal calcium channel TRPV5 and potentially is protective against calcium nephrolithiasis by increasing urinary calcium reabsorption^17^.

Since the high GC content in combination with the genomic architecture of the VNTR largely prevents direct sequencing, a probe extension (snapshot) assay refined by mass spectrometry validation has been developed as a first diagnostic test. The test needs to accomplish the detection of a single 60-mer unit carrying the insC against a high background of normal units (expected frequency between 0.8% (1/125 units) to 5% (1/20 units) depending on individual VNTR allele sizes)^4,5,18^. The evident limitations of the assay, the loss of almost all structural and positional information, led us to hypothesize on the existence of other causative alterations located within the VNTR that cannot be interrogated by the current assay.

To generate complete VNTR assemblies we thus adapted targeted single molecule real time (SMRT) sequencing on the PacBio RS II platform. We argued that this method could improve the testing in general and especially of sporadic cases, a group that has been largely omitted from current studies^4,5,19,20^. We show precise VNTR structural assembly and location of the causative insC, a prerequisite for identification of additional causative variants within the VNTR and detailed characterisation of genotype-phenotype correlations in ADTKD-*MUC1*.

## Material and Methods

### Probands

All investigations were conducted in accordance with the principles of the Declaration of Helsinki and after obtaining written informed consent. The study was approved by the local institutional review boards of the Universities of Cologne (IRB approval Nr 237/2013). Out of our (non-syndromal) ADTKD cohort previously tested negative for the ADTKD-genes *UMOD*, *REN* and *HNF1B* (including MLPA analysis for *HNF1B*, SALSA MLPA P241; MRC-Holland) we selected DNA samples from 9 families with positive snapshot findings as well as six families and one sporadic case with negative snapshot results who were still suspected to have ADTKD-*MUC1* based on their clinical course and kidney biopsy findings for SMRT sequencing. Clinical and biochemical data were collected retrospectively from medical charts.

### MUC1 VNTR PCR

Proband DNA was extracted from blood samples by NaCl precipitation and 30ng genomic DNA were used as templates^21^. The PCR protocol was adapted from a previous study with slightly modified cycling conditions and PCR composition^22^. Custom-designed 16 bp barcodes were added to the PCR primers for multiplexing different individuals following the guidelines for using PacBio Barcodes for SMRT Sequencing (Pacific Biosciences). Briefly, 25 µl reactions contained 30 ng of genomic DNA, 0.25 µM of PS2 (5′-XXXXXXXXXXXXXXXGGAGAAAAGGAGACTTCGGCTAC CCAG-3′; X stands for each nucleotide) and PS3 (5′-XXXXXXXXXXXX XXXXGCCGTTGTGCACCAGAGTAGAAGCTGA-3′) primers, 5% DMSO, 0.4 µM dNTP´s, 1 × reaction buffer with 1.5 mM MgCl_2_, 250 µM MgCl_2_, and 0.9 U DyNAzyme EXT DNA polymerase (Finnzymes, GRI Research, Braintree, UK). Thermocycling on C1000 touch instruments (Bio-Rad) included initial denaturation (1 min 30 s at 96°), 24 cycles (40 s at 96°, 30 s at 65° and 6 min at 68°) and final extension (10 min at 68°). The reaction was conducted three times for each sample, which were subsequently pooled. Amplification of VNTR (25 µl of each pooled sample) was verified by agarose gel electrophoresis (0.7%). The GeneRuler DNA Ladder (1 kb, 0.5 µg/µl, Fermentas) was used as a size marker. The remaining PCR products (50 µl) were sorted by allele length: (<4000 bp (≤66 variable repeat units) or >4000 bp (≥66 variable repeat units)), diluted 1:2 with H_2_O and purified using AMPure beads (Agencourt) according to the manufacturer´s instructions. AMPure bead:DNA ratio was adapted to the length of the allele of interest. Molar concentration was verified using the Qubit 3.0 Fluorometric Quantitation system (ThermoFischer).

### Library preparation and SMRT sequencing

Target amplicons of equal size were pooled equimolar and a SMRT bell library was prepared as recommended by Pacific Biosciences (10 kb Template Preparation and Sequencing with Low-Input DNA) without an initial fragmentation. Raw sequence data is available upon request. Libraries were quantified by fluorometry and quality was assessed by capillary electrophoresis (Agilent DNA 12000 reagents and chips, Agilent). SMRT bell templates were bound to polymerases using the DNA/polymerase binding kit P6 and v2 primers. Polymerase-template complexes were bound to magnetic beads with the Magbead Binding Kit and sequencing was done on the PacBio RS II sequencer with C4 sequencing reagents with a movie length of 180 or 360 min. Cluster analysis to extract haplotypes of individuals was done with the in house algorithm (Wenzel and Pannes unpublished). Amplicons were split according to the 16mer barcodes of PacBio. Additionally amplicons were split with a Perl script.

### Assembly of the *MUC1* VNTR using an in-house algorithm

Assembly of both paternal VNTR alleles was performed with the help of an in-house developed algorithm using a database-software (Microsoft ACCESS). The barcode-separated reads were converted into text files using Notepad++ editor and loaded into the database. Each single read was recognized by “ > m” (fasta format) and at the end by barcodes, respectively. The second step included the identification of start and end of each single 60-mer repeat unit by known and universal sequences of fixed repeat units 1 and 9. Part three included the determination of the repeat type based on sequences of actual known repeat variants described in Kirby *et al*. Certain repeat types are recognized based on selected base pair sequences within one repeat unit. The 60 basepair sequences of all known repeat types were subdivided into 9 “identification sequences”, respectively, each with a length of 4 bases in a row. This procedure overcomes the sequence variation based on reading failures made by the polymerase. Reconstruction of the repeat type assembly can be transferred into Microsoft EXCEL including two sheets: 1. Identified repeat variants corresponding to the capital letter code provided by Kirby *et al*.^5^ are shown in the first sheet for every single read allowing complete VNTR assembly. If one repeat unit type cannot be identified this position remains blank providing the possibility to identify so far unknown repeat variants. 2. The second sheet contains the sequence of each single read including the identified repeat variant, respectively, allowing the verification of identified and not identified single repeat units by manual inspection. For each individual two different alleles were identified.

### Data Availability

The publicly accessible database listed below:

- National Center for Biotechnology Information (NCBI; http://ncbi.nlm.nih.gov/),
- UCSC Genome Bioinformatics (http://genome.ucsc.edu/),
- 1000 Genomes (http://1000genomes.org/) and
- Broad Institute (http://exac.broadinstitute.org).

The datasets generated during and/or analysed during the current study using the in-house algorithm are available from the corresponding author on reasonable request.

## Results

### Prerequisites for MUC1-VNTR long-read sequencing

**Figure 2:**
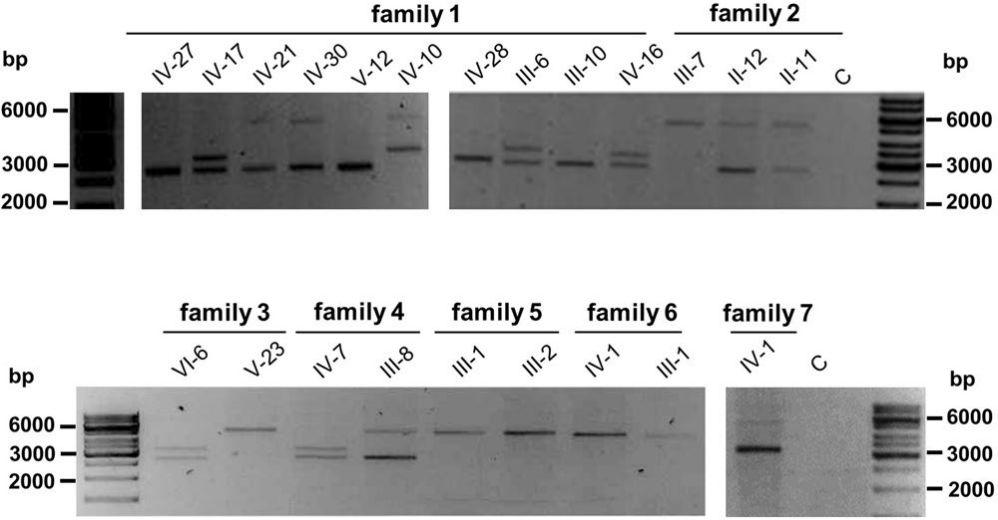
PCR amplification of the *MUC1*-VNTR. Depiction of amplicons spanning the *MUC1*-VNTR of families F1 to F7 separated by agarose gel electrophoresis (0.7% gel). Product sizes range between 2000 and 5000 bp. C, water control (see Table 1 and Fig. 4a).

Using a modified PCR protocol published by Fowler *et al*. allowed stable generation of PCR amplicons across a wide range of *MUC1* VNTR allele sizes (from 25 to 74 60-mer units corresponding to ca. 1.5 to 4.5 kb amplicons) that could be visualized by agarose gel electrophoresis (Fig. 2)^22^.

The fidelity of the PCR was validated by allele length determination using Southern blot analysis (data not shown).

We observed preferential amplification of shorter VNTR alleles (<4 kb), a phenomenom that is known as short allele dominance^23,24^. PCR amplification had to be repeated for 8 out of 43 individuals with at least one long allele, for PCR failure.

### Targeted SMRT sequencing of the *MUC1*-VNTR

Direct sequencing of the long-range *MUC1*-VNTR on the PacBio RS II platform was performed in 43 individuals (16 unaffected, 32 affacted) in 6 independent runs and resulted in an average sequencing depth of 160 complete reads (minimum 4, maximum 600 reads). Libraries were prepared by pooling 5 to 18 long-range (LR) PCR products depending on VNTR size that were subsequently analyzed on single SMRT cells.

Short allele dominance was observed in the first run. Shorter VNTR alleles (<4 kb) were overrepresented (range 32 to 600 complete reads) compared to longer alleles (range 4 to 229 complete reads). To minimize this effect PCR products were pooled for library preparation according to their length in all further runs into two libraries containing amplicons <4 and >4kb, respectively (for detailed information please see Material & Methods). This measure improved read depth for the longer alleles (range 36 to 440 complete reads).

Sequencing had to be repeated for 8 individuals for low complete VNTR coverage, which was mostly associated with allele size >4kb.

### *MUC1* VNTR SMRT sequencing in ADTKD families previously tested positive

**Figure 3:**
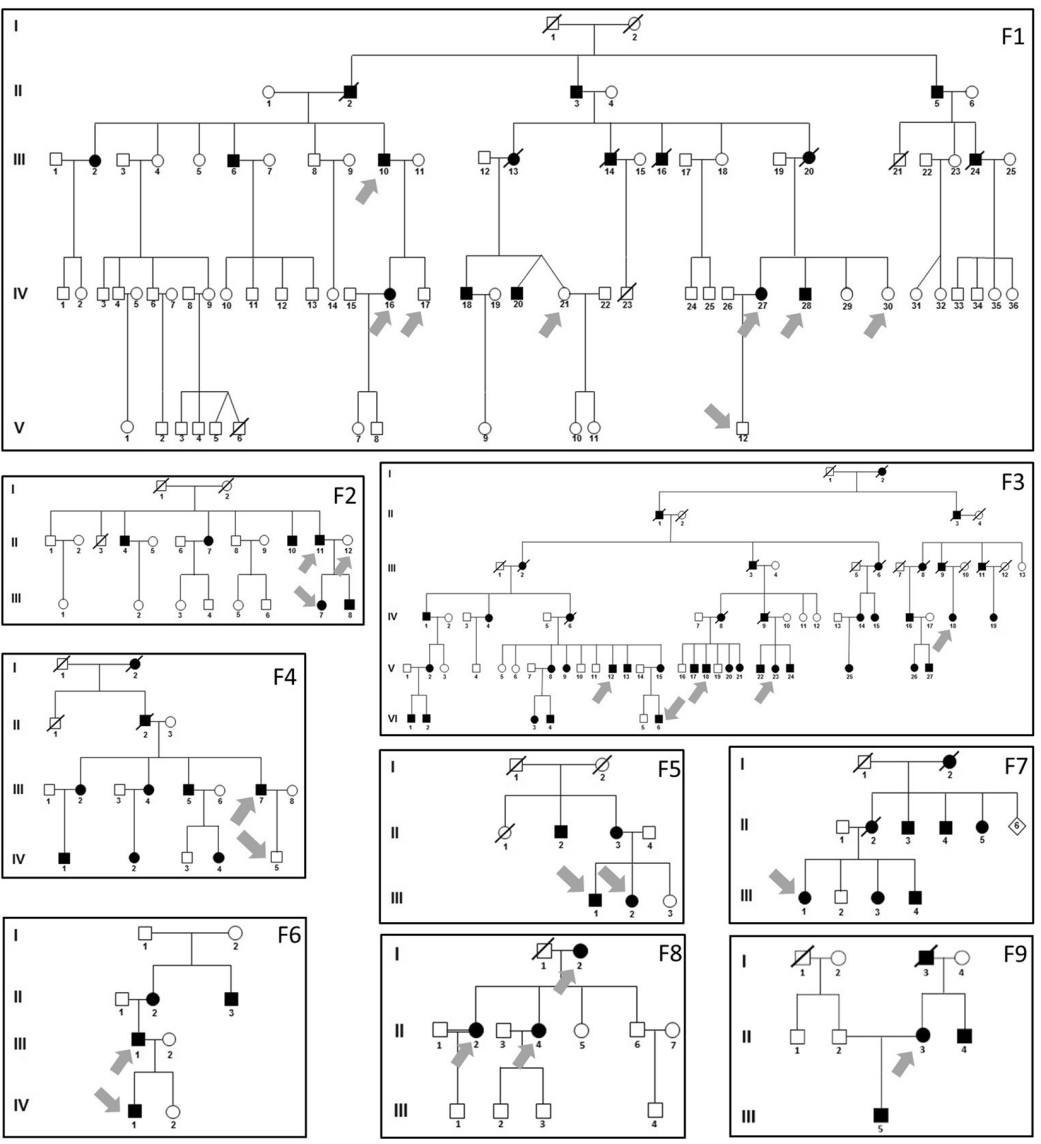
Families with ADTKD. Pedigrees of the nine InsC positive families. Black symbols indicate affected individuals, white symbols indicate unaffected status, and slashed symbols indicate deceased individuals. DNA for SMRT sequencing analysis was available from all individuals marked with arrows. Generations are numbered in roman numerals.

In all 9 families SMRT sequencing reconfirmed the insC previously detected by the probe extension assay with the exception of F7 in whom the insC was first identified by SMRT sequencing and later reconfirmed by the snapshot method (data not shown). In three families (F3*-F5*) the insC has been previously reported by Ekici *et al*., while the remaining six families (F1-F2, F6-F9) represent new families (Fig. 3)^4^.

### Interfamilial variability of insC position within the X-unit in the VNTR

SMRT sequencing consistently enabled us to generate sequence reads that allowed for complete assembly of both the wildtype and the ADTKD risk allele carrying the insC.

The precise location of the 60-mer unit location harbouring the insC within the *MUC1*-VNTR could be solved in eight of the nine positive families studied. In family F3 the insC was detected in three affected family members, but for unclear reasons we were not able to locate the precise 60-mer unit here.

**Figure 4:**
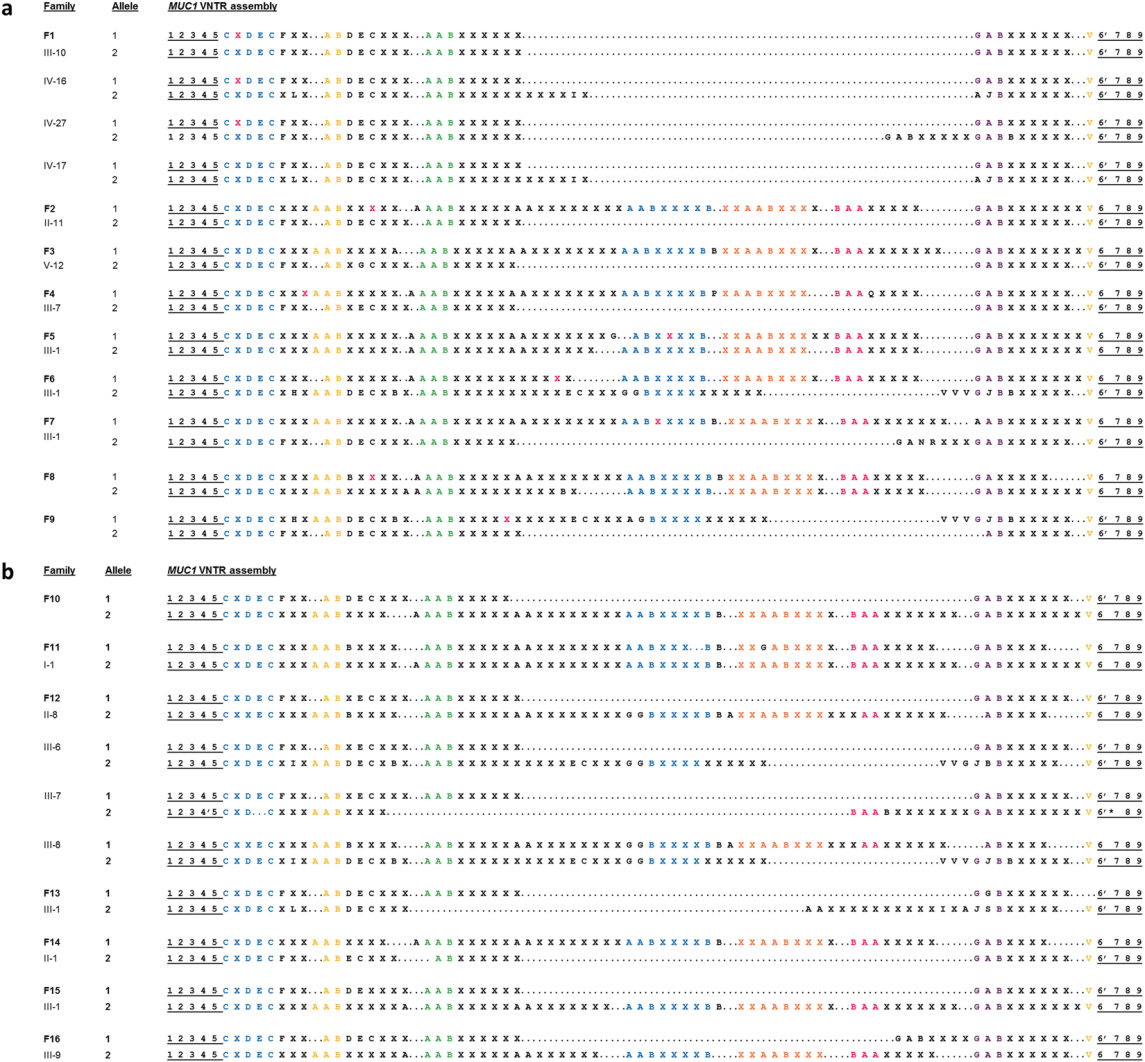
(**a**) Complete assembly of the risk and non-risk *MUC1*-VNTR alleles from positive tested families F1–9. For all families (F1–9), the structure of the risk *MUC1*-VNTR allele and the exact position of the single mutant repeat unit (with exception of family 3) and their sequence context were determined, respectively. Representative assembly of the VNTR as a series of 60mer units covering hg19 chr1 positions 155,160,963 to 155,162,030 (inclusive), and oriented relative to the *MUC1* coding strand (Human GRCH37/hg19; negative strand). Repeat units shown in red contain the insertion of an additional C into the seven C-stretch sequence appearing at relative positions 53–59 of a single repeat unit. Non-risk alleles are shown for all individuals. Uniform pseudo-repeat units 1–5 and 6–9 encompassing the variable repeat region are underlined. The assembly of the hypervariable VNTR is not arbitrary, but rather follows uniform patterns where certain unit stretches were conserved in all individuals between families regardless of the VNTR allele size. Uniform repeat type stretches are highlighted with different colours. (**b**) Complete assembly of both *MUC1*-VNTR alleles from negative tested families F10–16. For all families (F10–16) the structure of both *MUC1* VNTR alleles were determined. Exemplary depiction of VNTR assembly as a series of 60mer units covering hg19 chr1 positions 155,160,963 to 155,162,030 (inclusive), and oriented relative to the *MUC1* coding strand (hg 19 negative strand). Uniform pseudo-repeat units 1–5 and 6–9 encompassing the variable repeat region are underlined. Uniform repeat type stretches are highlighted with different colours. In the asymptomatic individual III-7 of F12 the fixed unit 6′ is lacking the last 18 bases (marked with an *) and unit 7 is completely missing.

Position of the insC ranged (counting only the variable units; see Fig. 4a, Table 1) from the second up to the 39^th^ unit and always occurred on the background of the X unit which is the most abundant 60-mer unit found within the VNTR (51.4–62.0%). The exact position of the causative variants was consistent in all affected individuals within families. Two families (F5 and F7) carried the insC in the 39^th^ variable unit, but their VNTR assembly and allele size was different (Table 1, Figs 3 and 4a). Of note the Swiss family F1 demonstrated exactly the same risk allele assembly and position of the prototypic mutation within the second X unit as one family (family 4) previously reported by Kirby *et al*.^5^ Identical assembly could indicate that these two families are related.

**Table 1:** Summary of 9 ADTKD-*MUC1* families with complete assembly of both *MUC1*-VNTR alleles tested positive by SMRT sequencing.

Family Origin	Individual sex	ADTKD status	Allele	Number variable repeat units	Insertion C in repeat unit number	VNTR Size (bp)	Affected SMRT pos/snapshot pos	Affected SMRT neg/snapshot neg	Number affected
F1 CHE							**4/4**	**3/3**	**≥20**
	III-10	affected	1	35	2	2641			
	male	ESRD (60y)	2	35	—	2640			
	IV-16	affected	1	35	2	2641 bp			
	female	CKD2 (39y)	2	41	—	3000 bp			
	IV-27	affected	1	35	2	2641			
	female	CKD3 (35y)	2	43	—	3120			
	IV-28	affected	1	35	2	2641			
	male	CKD2–3 (46y)	2	36	—	2700			
	IV-17	unaffected	1	35	—	2640			
	male	(37y)	2	41	—	3000			
	IV-21	unaffected	1	35	—	2640			
	female	(52y)	2	73	—	4920			
	V-12	unaffected	1	35	—	2640			
	male	(18y)	2	43	—	3120			
F2 GER							2/2	1/1	≥7
	II-11	affected	1	71	14	4801			
	male	ESRD (32y)	2	35	—	2640			
	III-7	affected	1	71	14	4801			
	female	CKD1–2 (29y)	2	73	—	4920			
	II-12	unaffected	1	35	—	2640			
	female	(63y)	2	73	—	4920			
F3^*^ GER							3/3	1/1	≥40
	V-12	affected	1	73	ambiguous	4921			
	male	ESRD (41y)	2	35	—	2640 bp			
	V-23	affected	1	73	ambiguous position	4921 bp			
	female	ESRD (43y)	2	71	—	4800			
	V-18	affected	1	73	ambiguous position	4921			
	male	ND	2	25	—	2040			
	VI-6	unaffected	1	41	—	3000			
	male	(44y)	2	34	—	2580			
F4* GER							1/1	1/1	≥9
	III-8	affected	1	70	8	4741			
		ESRD (41y)	2	35	—	2640			
	IV-7	unaffected	1	42	—	3060			
		(39y)	2	35	—	2640			
F5* GER							2/2	0/0	4
	III-1	affected	1	71	39	4801			
	male	ESRD (43y)	2	67	—	4560			
	III-2	affected	1	71	39	4801			
	female	CKD3 (40y)	2	67	—	4560			
F6 GER							2/2	0/0	
	III-1	affected	1	66	29	4501			
	male	ESRD (ND)	2	61	—	4200			
	IV-1	affected	1	66	29	4501			
	male	CKD3 (33y)	2	66	—	4500			
F7 NLD							1/1	0/0	≥8
	III-1	affected	1	74	39	4981			
	female	ESRD (49y)	2	42	—	3060			
F8 TUR							3/3	0/0	≥4
	II-7	affected	1	69	14	4681			
	female	ESRD (60y)	2	68	—	4560			
	III-5	affected	1	69	14	4681			
	female	ESRD (39y)	2	34	—	2580			
	III-6	affected	1	69	14	4681			
	female	CKD3 (37y)	2	34	—	2580			
	?	unaffected	1	35		2640			
	male	(30y)	2	68		4620			
	?	unaffected	1	34		2580			
	male	(38y)	2	72		4860			
F9 GER							1/1	0/0	3
	II-2	affected	1	34	24	2581			
	female	ESRD (44y)	2	60	—	4140			

^*^Indicates that families F*3 to F*6 have been previously reported^4^.

F, family; CKD, chronic kidney disease stage and age at CKD stage in years; ESRD, endstage renal disease and age at ESRD in years; KTx, kidney transplantation; ND, no data available; CHE, Switzerland; GER, Germany; NLD, Netherlands; TUR, Turkey; neg, negative; pos, positive; y, years.

### SMRT sequencing in unclear non UMOD/HNF1B/REN ADTKD families

To further investigate our hypothesis that the VNTR might be a mutational hotspot prone to other mutations (e.g. small deletions etc.) within or outside of the 7C homopolymer a small cohort of three large families (F12, F13, and F16), three smaller families (F10, F14 and F15) and one sporadic case (F11), all compatible with a diagnosis of ADTKD-*MUC1*, were analysed (Table 2, Fig. 4b).

**Table 2:** Summary of the 7 suspected ADTKD-*MUC1* families (previously tested negative for *UMOD*, *REN*, *HNF1B* and *MUC1* by the snapshot method) with unremarkable SMRT sequencing results, despite complete assembly of both VNTR alleles.

Family origin	Individual sex	ADTKD status	Allele	Number variable repeat units	Insertion C in repeat unit number	VNTR size (bp)	Affected SMRT pos/snapshot pos	Affected SMRT neg/snapshot neg	Number affected
F10 GER							0/0	1/1	≥4
		affected	1	34	—	2580			
	male	ESRD (55y)	2	73	—	4920			
F11 GER							0/0	1/1	1
	I-1	affected	1	68	—	4620			
	female	ESRD (xxy)	2	74	—	4980			
F12 GER							0/0	3/3	≥8
	II-6	affected	1	35	—	2640			
	female		2	37	—	2760			
	II-8								
	affected	1	35	—	2640				
	female	CKD 3 (60y)	2	70	—	4740			
	III-4	affected	1	35	—	2640			
	male		2	21	—	1800			
	III-5	affected	1	35	—	2640			
	male		2	21	—	1800			
	III-6								
	affected	1	35	—	2640				
	female	ESRD (27y)	2	61	—	4200			
	III-7	affected							
	1	35	—	2640					
	male	CKD 3 (26y)	2	36	—	2700			
	II-7	unaffected	1	36	—	2700			
	male		2	61	—	4200			
	III-8	unaffected	1	61	—	4200			
	female	(24y)	2	70	—	4740			
F13 NDL							0/0	1/1	≥6
	III-1	affected	1	34	—	2580			
	female	ESRD (41y)	2	40					
	—	2940							
F14 GER							0/0	1/1	≥2
	II-1	affected							
	1	68	—	4620					
	female	ERSD (43y)	2	33	—	2520			
F15 CHE							0/0	1/1	2
	III-1	affected	1	35	—	2640			
	male	CKD 3 (44y)	2	73	—	4920			
	II-2	affected	1	35	—	2640			
	female		2	57	—	3960			
					—				
	II-1	unaffected	1	66	—	4500			
	?		2	73	—	4920			
F16 NDL							0/0	1/1	≥18
	III-9	affected	1	33	—	2520			
	male	CKD2–3 (66y)	2	65	—	4250			

F, family; CKD, chronic kidney disease stage and age at CKD stage in years; ESRD, endstage renal disease and age at ESRD in years; KTx, kidney transplantation; ND, no data available; CHE, Switzerland; GER, Germany; NDL, Netherlands; TUR, Turkey; neg, negative; pos, positive; y, years.

No other small insertion or deletion resulting in a frameshift consequence and no causative structural variant could be detected by complete VNTR assembly, although family F12 demonstrated linkage to a 12.9 Mb interval encompassing the *MUC1*-locus on chromosome 1q21 with a maximum parametric LOD score of 1.8 under assumption of autosomal dominant inheritance (data not shown; only affected individuals analyzed)^25^. No linkage data were available of the latter two larger families F13 and F16. Large deletions and loss of heterozygosity (LOH) could be excluded in this cohort as we were always able to reconstruct two parental VNTR alleles.

*MUC1* mutations outside the VNTR were excluded by Sanger sequencing in all families (plus whole exome sequencing in F12).

### Identification of novel repeat variants

**Figure 5:**
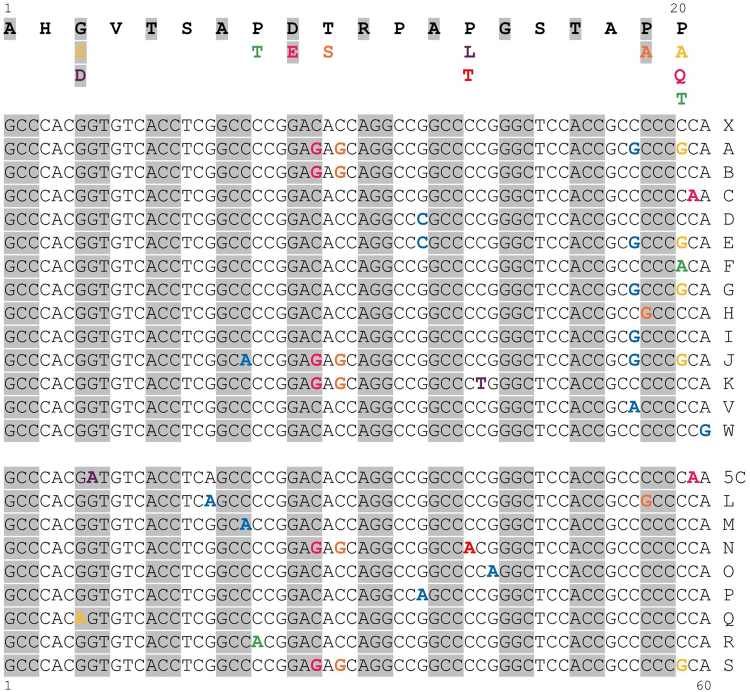
Novel repeat variants. SMRT sequencing revealed nine novel repeat (noncausative) variants. Initially detected at the protein level, variant sequences were later defined with respect to their topology in peripheral regions of the VNTR domain^30–32^. According to these studies, the flanking regions were characterized by a highly conserved pattern of variant units A-F in genomic DNA from individuals of different ethnic background. It was concluded that the variant repeat topology could have resulted from multiple duplication events in the phylogeny of the *MUC1* repeat domain. Later it was shown that the entire domain contains clusters of variant A and B units interspersed in clusters of X units^30^. As the A, C, E, and F unit exhibit replacements of the second proline residue in the STAPPA motif, the expressed mucin is characterized by a pronounced under-glycosylation here. Again at the protein level the DT to ES replacement within the immunodominant DTR motif results in higher conformational flexibility at this site and altered B lymphocyte responses^32^. Upper case letters were continued following the one letter code introduced by Kirby *et al*. Nucleotide sequence differences to the canonical repeat variant “X“ are highlighted in dark grey. The repeat type 5C is a fusion of repeat types 5 and C. It is composed of the first half of the uniform repeat type 5 and the second half of the repeat type C. At the top corresponding amino acid sequences are shown for the canonical and variant-containing units. Synonymous variants are marked in light blue. Nonsynonymous variants and corresponding amino acid exchanges are highlighted in different colors. Among other variants we identified two novel non-synonymous variants in repeat units N and Q resulting in amino acid replacements G to S in the HGT motif (CCG to ACG in unit Q) and P to T in the PAP motif (CCG to ACG in unit N).

We found altogether nine novel repeat variants and annotated them in continuation of the one letter code introduced by Kirby *et al*. (Fig. 5) since there is no suitable HGVS nomenclature for complex tandem repeat (TR). Three of these repeat variants include the following non-synonymous SNPs on relative repeat position 7G > A (p.G3S), position 22C > A (p.P8T), position 40C > A (p.P14T) termed Q, R, and N. In addition three novel repeat variants with synonymous SNPs were found, which were termed L (rel. position 18G > A, p.P6=), O (rel. position 42G > A, p.P6=), and P (36G > A, p.P6=). The remaining repeat variants 5C, M and S consist of previously described SNPs in a novel combination. Variant 5C is a fusion of previously described variants 5 (fixed variant) and C (non-synonymous SNPs at rel. positions 8G > A (p.G3D) and 59C > A (p.P20Q)). The repeat variant M contains one synonymous SNP at rel. position 21C > A (p.P7=) and variant S three non-synonymous known SNP´s at relative positions 27C > G (p.D9E), 29C > G (p.T10S), and 58C > G (p.P20A).

## Discussion

Sequencing with the PacBio RS II system based on single molecule real time (SMRT) technology offers the benefit of extraordinarily long sequencing reads with high accuracy. We adapted SMRT for targeted sequencing of the mutational hotspot in the coding VNTR of the *MUC1* gene. The need for double stranded DNA and the comparatively low capacity of the system made the generation of PCR amplicons spanning the VNTR indispensable. SMRT sequencing has been previously performed for complex genomic regions, including another mucin, encoded by the *MUC5AC* gene, mainly secreted in the respiratory tract^26^.

The discovery of the genetic basis of this long sought ADTKD type cannot be overemphasized as it has opened the field of tandem repeats to the spectrum of hereditary kidney disease. The paper by Kirby *et al*. also points out to the pitfall of missing hereditary disease contained in those repetitive sequences with the current strategies employed in gene identification studies^5^.

Tandem repeats (TR) are a major class of repetitive DNA which makes up for a significant amount of the humane genome. TR the size of 9 to 80 bp that are repeated a few to more than a hundred times are also called VNTRs as they can be highly polymorphic within a population. From a molecular point of view tandem repeat polymorphisms (TRPs) provide a dynamic source of genomic variability as their mutation rate is higher and the extent of polymorphism (multiallelic) is more diverse than that contained in single nucleotide polymorphisms (SNPs). Expansions in short/simple TR´s are known to cause many monogenic disorders, which mainly result in neurodegenerative disease.

The technical dilemma caused by the difficult nature of the VNTR’s genomic sequence (tertiary structure resulting from the repetitive sequence, GC content, and repeat length) is well illustrated by the surprisingly few publications on ADTKD-*MUC1* after the initial description. These inherent problems have created a diagnostic bottleneck for *MUC1* testing worldwide with only a few laboratories performing the probe extension assay and only one laboratory being able to validate the snapshot assay by mass spectrometry^4,5,20,27^.

SMRT sequencing allowed complete assembly of both the wildtype and the ADTKD risk allele and precise positioning of the insC in all families (except family F3). In contrast even mutations occurring at the 5′ and 3′ borders of the VNTR are usually not accessible to Sanger sequencing or to short read based NGS technologies.

The reason why several affected family members from F3 repeatedly showed ambiguous position of the insC on various X units remains unexplained, but could not be related to a low number of complete VNTR reads. Interestingly, the prototypic mutation insC was exclusively found on the background of the X unit in our cohort. In contrast the only other publication providing data on VNTR assembly identified the insC on the variable X- and B- unit and on the”fixed” 5-unit^5^. Our observation could be an accidental finding as the number of analysed families in our study was still small and the X unit constitutes the most abundant unit within the VNTR. A binomial test calculates a one-tailed P-value of 0.016 for the chance of exactly 8 mutational hits in the X unit in 8 families, which does not support the hypothesis of a chance finding.

By pinpointing the precise repeat unit carrying the prototypic insC we could confirm the allelic heterogeneity of *MUC1* associated kidney disease. The mutational spectrum is further expanded by a recent study that identified the first causative *MUC1* mutation close to but outside of the VNTR that is accessible to standard sequencing technology and an abstract of Morniere *et al*. who reported the presence of a new five base pair deletion within the VNTR identified by the probe extension assay in one of 15 families tested positive. It would be interesting to see SMRT-sequencing results of the latter family^28,29^.

In the largest study on ADTKD-*MUC1* 24 families, with the insC identified by the snapshot method, have been described with high intra- and interfamilial variability regarding the progression of renal disease and onset of ESRD^20^. They speculated on the existence of anticipation (worsening of the phenotype in succeeding generations) in some families, a phenomenon that has been noted in the very first publication on the *MUC1* locus on chromosome 1q21^25^. Although the underlying molecular mechanism in a non-expansion TR would be unclear at the moment, precise structural information on the *MUC1*-VNTR (allele sizes, position of the frameshift mutation, polymorphisms that could possibly alter the structure and thereby the toxicity of the frameshift protein) could provide valuable information to predict a potential role of the hypervariable VNTR in anticipation/disease progression from observer bias. To our knowledge this study contains the most comprehensive data on *MUC1*-VNTR topology, but was neither designed to nor is powered to answer those questions. Assessing the impact of coding and noncoding variation for potential genotype phenotype correlations in ADTKD-*MUC1* will require a larger number of resolved VNTR datasets. Thus, our study provides a methodological basis to sequence this region which might help to uncover the factors explaining the considerable intra- and interfamilial variability in progression of *MUC1* related kidney disease.

As the pathomechanisms in ADTKD-*MUC1* are likely linked to inflammatory and immune processes in the kidney tissue and structural variation in the VNTR domain has been shown to alter B lymphocyte response, it is not beyond reason to assume a potential role of the VNTR’s topology here (please see Fig. 5 for further information)^30–32^.

In summary we recommend to perform first line or complementary SMRT sequencing in all snapshot positive and negative suspected ADTKD-*MUC1* cases (to potentially identify novel mutations occurring within the VNTR in the latter). We propose to document next to the precise position of the causative mutation, allele lengths and (sequence) topology of both the wild type and the disease causing allele in all future studies until the role of the VNTR becomes clearer.

## References

[1] Eckardt K-U (2015). Autosomal dominant tubulointerstitial kidney disease: diagnosis, classification, and management–A KDIGO consensus report. Kidney Int..

[2] Dahan K (2001). Familial juvenile hyperuricemic nephropathy and autosomal dominant medullary cystic kidney disease type 2: two facets of the same disease?. J. Am. Soc. Nephrol..

[3] Bollée G (2011). Phenotype and outcome in hereditary tubulointerstitial nephritis secondary to UMOD mutations. Clin J Am Soc Nephrol.

[4] Ekici AB (2014). Renal fibrosis is the common feature of autosomal dominant tubulointerstitial kidney diseases caused by mutations in mucin 1 or uromodulin. Kidney Int..

[5] Kirby A (2013). Mutations causing medullary cystic kidney disease type 1 lie in a large VNTR in MUC1 missed by massively parallel sequencing. Nat. Genet..

[6] Hart TC (2002). Mutations of the UMOD gene are responsible for medullary cystic kidney disease 2 and familial juvenile hyperuricaemic nephropathy. J. Med. Genet..

[7] Zivná M (2009). Dominant renin gene mutations associated with early-onset hyperuricemia, anemia, and chronic kidney failure. Am. J. Hum. Genet..

[8] Lindner TH (1999). A novel syndrome of diabetes mellitus, renal dysfunction and genital malformation associated with a partial deletion of the pseudo-POU domain of hepatocyte nuclear factor-1beta. Hum. Mol. Genet..

[9] Faguer S (2011). Diagnosis, management, and prognosis of HNF1B nephropathy in adulthood. Kidney Int..

[10] A novel gene containing a trinucleotide repeat that is expanded and unstable on Huntington’s disease chromosomes. The Huntington’s Disease Collaborative Research Group. *Cell* **72**, 971–983 (1993). 10.1016/0092-8674(93)90585-e 8458085

[11] Kremer EJ (1991). Mapping of DNA instability at the fragile X to a trinucleotide repeat sequence p(CCG)n. Science.

[12] Gendler SJ (1990). Molecular cloning and expression of human tumor-associated polymorphic epithelial mucin. J. Biol. Chem..

[13] Swallow DM (1987). The hypervariable gene locus PUM, which codes for the tumour associated epithelial mucins, is located on chromosome 1, within the region 1q21-24. Ann. Hum. Genet..

[14] Fanni D (2012). MUC1 marks collecting tubules, renal vesicles, comma- and S-shaped bodies in human developing kidney tubules, renal vesicles, comma- and s-shaped bodies in human kidney. Eur J Histochem.

[15] Leroy X (2002). Expression of human mucin genes in normal kidney and renal cell carcinoma. Histopathology.

[16] Silva F (2001). MUC1 gene polymorphism in the gastric carcinogenesis pathway. Eur. J. Hum. Genet..

[17] Nie M (2016). Mucin-1 Increases Renal TRPV5 Activity *In Vitro*, and Urinary Level Associates with Calcium Nephrolithiasis in Patients. J. Am. Soc. Nephrol..

[18] Blumenstiel B (2016). Development and Validation of a Mass Spectrometry-Based Assay for the Molecular Diagnosis of Mucin-1 Kidney Disease. J Mol Diagn.

[19] Huddleston J (2014). Reconstructing complex regions of genomes using long-read sequencing technology. Genome Res..

[20] Bleyer AJ (2014). Variable clinical presentation of an MUC1 mutation causing medullary cystic kidney disease type 1. Clin J Am Soc Nephrol.

[21] Miller SA, Dykes DD, Polesky HF (1988). A simple salting out procedure for extracting DNA from human nucleated cells. Nucleic Acids Res..

[22] Fowler JC, Teixeira AS, Vinall LE, Swallow DM (2003). Hypervariability of the membrane-associated mucin and cancer marker MUC1. Hum. Genet..

[23] Walsh PS, Erlich HA, Higuchi R (1992). Preferential PCR amplification of alleles: mechanisms and solutions. PCR Methods Appl..

[24] Wattier R, Engel CR, Saumitou-Laprade P, Valero M (1998). Short allele dominance as a source of heterozygote deficiency at microsatellite loci: experimental evidence at the dinucleotide locus Gv1CT in Gracilaria gracilis (Rhodophyta). Molecular Ecology.

[25] Christodoulou K (1998). Chromosome 1 localization of a gene for autosomal dominant medullary cystic kidney disease. Hum. Mol. Genet..

[26] Guo X (2014). Genome reference and sequence variation in the large repetitive central exon of human MUC5AC. Am. J. Respir. Cell Mol. Biol..

[27] Musetti C (2016). Testing for the cytosine insertion in the VNTR of the MUC1 gene in a cohort of Italian patients with autosomal dominant tubulointerstitial kidney disease. J Nephrol..

[28] Yamamoto, S. *et al*. Analysis of an ADTKD family with a novel frameshift mutation in MUC1 reveals characteristic features of mutant MUC1 protein. *Nephrol Dial Transplant*, 10.1093/ndt/gfx083 10.1093/ndt/gfx083 29156055

[29] Morinière, V. *et al*. Mutations in a large VNTR of MUC1 are frequent in autosomal dominant medullary cycstic kidney disease (MCKD). *J. Am. Soc. Nephrol*. **24** (2013).

[30] Engelmann K, Baldus SE, Hanisch FG (2001). Identification and topology of variant sequences within individual repeat domains of the human epithelial tumor mucin MUC1. J. Biol. Chem..

[31] Müller S (1999). High density O-glycosylation on tandem repeat peptide from secretory MUC1 of T47D breast cancer cells. J. Biol. Chem..

[32] von Mensdorff-Pouilly S (2005). Sequence-variant repeats of MUC1 show higher conformational flexibility, are less densely O-glycosylated and induce differential B lymphocyte responses. Glycobiology.

